# La survie en hémodialyse chronique au Cameroun

**DOI:** 10.11604/pamj.2017.26.97.9658

**Published:** 2017-02-24

**Authors:** Hermine Fouda, Gloria Ashuntantang, François Kaze, Marie-Patrice Halle

**Affiliations:** 1 Faculté de Médecine et de Science Biomédicales de Yaoundé, Cameroun; 2 Faculté de Médecine et des Sciences Pharmaceutiques de Douala, Cameroun

**Keywords:** Hémodialyse, mortalité, survie, Hemodialysis, mortality, survival

## Abstract

**Introduction:**

L'hémodialyse est le seul traitement de substitution rénale disponible au Cameroun; elle est subventionnée à 95% par l'Etat depuis 2002 et le nombre de centre de dialyse va croissant. Cependant, depuis l'ouverture du premier centre en 1990, aucune donnée n'existe sur la survie des hémodialysés chroniques.

**Méthodes:**

Nous avons conduit une étude de cohorte prospective multicentrique de 15 mois dans le but d'évaluer la mortalité et les facteurs qui influencent la survie des hémodialysés chroniques camerounais.

**Résultats:**

Nous avons suivi 197 patients dont 109 incidents. L'âge moyen était de 47,97± 13,19 ans et 55% étaient de sexe masculin. La durée moyenne en dialyse des patients prévalent était de 12,5 mois. Le taux de mortalité était de 57,58% dont 50% pendant les 3 premiers mois et le taux d'abandon était de 8,6%. L'urémie et les sepsis sur cathéter étaient les principales causes de décès. La survie globale à 15 mois était de 30,77%, avec une durée moyenne de vie de 8 mois. Les patients prévalent, la résidence dans la ville du centre de dialyse, la prise en charge non familiale, le suivi pré dialyse > 3 mois, la cholestérolémie à l'entrée en dialyse> 1,5g/l, un score mental > 25 étaient associés à une meilleure survie.

**Conclusion:**

La mortalité en hémodialyse au Cameroun est élevée, avec une survie moyenne de 8 mois et la plupart des patients décèdent au cours des 3 premiers mois.

## Abstract (en)

**Introduction:**

Haemodialysis is the only renal replacement therapy available in Cameroon; 95% of patients has received subsidies by the State since 2002 and the number of dialysis centers is growing. However, since the opening of the first center in 1990, there are no data on survival in chronic dialysis patients.

**Methods:**

We conducted a multicenter prospective cohort study of 15 months in order to assess mortality rates and factors that influence the survival of chronic hemodialysis patients in Cameroon.

**Résults:**

We followed 197 patients of whom 109 were incident patients. The average age was 47,97± 13.19 years and 55% were male. The mean duration of dialysis in prevalent patients was 12.5 months. The mortality rate was 57,58%, 50% of which occurred the first 3 months and abandonment rate was 8.6%. Uremia and catheter-related sepsis were the main causes of death. At a median follow up of 15 months, the overall survival was 30.77%, with an average duration of life of 8 months. Prevalent patients, patient’s place of residence where the dialysis center is located, nonfamily management, predialysis follow up > 3 months, blood cholesterol when patient first started on dialysis> 1.5g/l, a mental test score > 25 were associated with a better survival.

**Conclusion:**

In Cameroon, mortality rate in hemodialysis patients is high, with a mean survival time of 8 months; most patients die within the first 3 months.

## Introduction

L'insuffisance rénale chronique terminale (IRCT) constitue un problème majeur de santé publique dans le monde de par sa prévalence, sa mortalité élevée et les coûts de sa prise en charge. En 2012, l'IRCT était considérée comme la maladie chronique la plus couteuse aux Etats-Unis représentant 5% de budget de santé globale pour moins de 1% de la population [1]. Dans les pays en voie de développement, et notamment en Afrique Subsaharienne, la mortalité liée à l'IRC serait élevée du fait de l'insuffisance des ressources matérielles, financières et humaines [2]. Au Cameroun, l'hémodialyse est le seul traitement de substitution rénale disponible. Avant, 2002, un seul centre d'hémodialyse existait et le coût de la dialyse variait entre 60-100 000 F CFA (120-200$), ce qui limitait l'accessibilité du traitement de l'IRCT. Depuis 2002, elle est subventionnée à 95% par l'Etat ramenant la séance de dialyse à 5000 FCFA (10$). De plus, l'Etat a facilité l'accès des patients à ce traitement par la création de nouveaux centres de dialyse dont le total s'élève à 10 en 2015. Ces mesures ont permis d'améliorer l'accessibilité au soin et d'accroitre le nombre de patients en hémodialyse chronique. Cependant aucune donnée n'existe sur la survie de ces patients. Le but de notre étude était d'évaluer la mortalité et les facteurs qui influencent la survie des hémodialysés chronique au Cameroun entre 2004 et 2005.

## Méthodes

Il s'agissait d'une étude de cohorte prospective multicentrique de 15 mois (août 2004 à octobre 2005) incluant tous les patients hémodialysés chroniques au Cameroun (hôpitaux généraux de Yaoundé et Douala, clinique 'le Métropolitain'): Le centre d'hémodialyse de l'hôpital général de Douala est le centre le plus ancien du Cameroun, ouvert depuis 1990. En 2004, il comportait 8 postes de dialyse, dont la majorité était de marque Fresenius, avec 3 médecins dont 1 néphrologue. Le centre d'hémodialyse de l'hôpital général de Yaoundé a ouvert en 2002; en 2004 il comportait 8 postes de marques Gambro et Fresenius, avec 2 néphrologues. Les centres d'hémodialyses des hôpitaux généraux de Douala et Yaoundé sont des centres publiques dans lesquels la dialyse est subventionnée par l'Etat, le coût d'une séance de dialyse est ainsi de 5000 FCFA (10$). Le centre d'hémodialyse de la clinique le Métropolitain est un centre privé, non subventionné par l'Etat et le coût d'une séance de dialyse est de 60000FCFA (120$). En 2004, il comportait 2 postes de marque Gambro avec un néphrologue. Un consentement éclairé des patients était obtenu avant leur inclusion. Les données sociodémographiques (âge, sexe, emploi, lieu de résidence) et économiques (revenu mensuel moyen, prise en charge des soins), les paramètres paracliniques (azotémie, créatininémie, calcémie, phosphorémie, hémoglobine, cholestérol total, cholestérol LDL, cholestérol HDL, triglycéride, antigène Hbs, anticorps HCV, sérologie HIV) à l'entrée en dialyse des patients incidents et la survenue d'infection, d'hospitalisation ou du décès en dialyse ont été collectés. La qualité de vie du patient a été évaluée à l'aide du Short Form 36 (SF36) et réalisé au moins 4 semaines après le début de la dialyse. Les comorbidités à l'entrée en dialyse étaient appréciées par l'indice de Charlson. Les définitions suivantes ont été utilisées pour les termes : adhérence en dialyse : elle était évaluée par la régularité aux séances de dialyse sur une période de 8 semaines. Elle était considérée comme mauvaise en cas d'absence volontaire à au moins 2 séances de dialyse sur cette période et bonne dans le cas contraire (< 2 absences sur 8 semaines). Anémie : elle était définie par un taux d'hémoglobine < 10g/dl. Accident vasculaire cérébral : survenue brutale d'un déficit moteur ± trouble de la conscience ± convulsions avec TA élevée, dans un contexte fébrile, avec ou sans confirmation scannographique. Compliance au suivi néphrologique en pré dialyse : était évaluée par 2 critères: la régularité au rendez-vous et la présence d'une FAV à l'entrée en dialyse. Selon la présence de 0, 1 ou 2 critères, elle était classée en mauvaise, médiocre ou bonne. Courte durée suivi néphrologique : durée suivi néphrologique en pré dialyse < 3 mois Dialyse programmée / non programmée: la dialyse était dite programmée, si la première dialyse est réalisée sur une FAV; et non programmée si elle est réalisée sur cathéter temporaire. Infection de cathéter: elle était définie par la présence d'une température > 37°C chez un patient porteur d'un cathéter et/ou de la présence de signes locaux d'infection (suppuration, signes inflammatoires) avec ou sans preuve bactériologique. Transfert tardif: transfert en néphrologie avec DFGe < 30 ml/min. L'urémie: réapparition des signes d'urémie chronique et/ ou survenue d'une ascite ou d'une péricardite sans autres étiologie retrouvées (notamment tuberculose) Patient incident: patient débutant l'hémodialyse pendant la période d'étude Patients prévalent: patients qui étaient déjà en hémodialyse au début de la période d'étude durant la période d'étude, 111 hémodialysés chroniques étaient enregistrés à l'hôpital général de Yaoundé, 89 à l'hôpital général de Douala et 1 à la clinique le Métropolitain. Les données des patients ayant des dossiers médicaux non retrouvés ou incomplets, celles des patients entrés en dialyse et décédés avant remplissage du questionnaire et celles des patients perdus de vu ou ayant immigré dans un autre pays ont été analysés. Les données ont été analysées avec le logiciel SPSS 11.0. Les tests de χ^2^, de Student, la formule de Kaplan et Meier, les tests de corrélation et de Log Rank ont été utilisés pour l'analyse statistique. Le seuil de significativité était p < 0,05.

## Résultats

**Figure 1:**
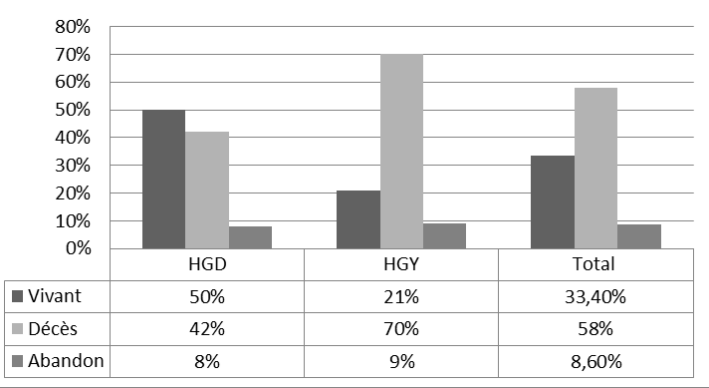
Répartition selon le devenir des patients, description du devenir des patients (décès, vivant, abandon) en fonction des hôpitaux

**Figure 2:**
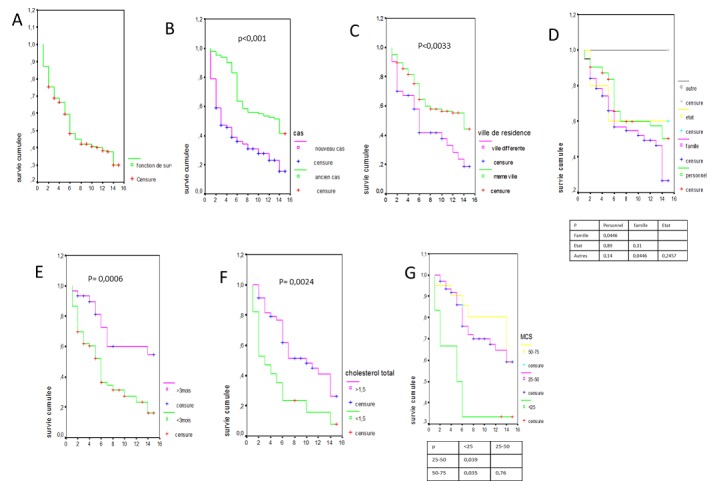
Courbes de survie A) description des courbes de survie, survie globale; B) survie en fonction de l’ancienneté en dialyse (nouveau cas= patient incident, ancien cas= patient prévalent); C) survie en fonction de la ville de résidence; D) survie en fonction du type de prise en charge financière; E) survie en fonction de la durée du suivi prédialyse; F): survie en fonction du taux de cholestérol total à l’entrée en dialyse; G) survie en fonction des scores de qualité de vie mentale

Au total, 197 patients ont été inclus dans notre étude, avec 109 patients incidents (55%) et 1 patient a été exclu. La durée moyenne en dialyse des patients prévalents était de 12,5 mois. Les données sociodémographiques, cliniques et paracliniques des patients à l'entrée en dialyse sont décrites dans les Tableau 1 et Tableau 2. Malgré la prescription de 4 heures de dialyse deux fois par semaine, le nombre moyen d'heure de dialyse effective par semaine était de 6,62 heures et 51,4% des patients avaient une mauvaise adhérence au traitement par hémodialyse. La majorité des patients n'adhéraient pas au traitement (51,4%) et ce manque d'adhérence était plus fréquent chez les patients qui résidaient hors de la ville du centre de dialyse (70,4% contre 45,1% p=0,023) L'évaluation de la qualité de vie des patients après la mise en dialyse montrait une mauvaise qualité de vie avec un Physical Component Summary Scale (PCS) score de 34,21 et un Mental Component Summary Scale (MCS) score de 41,03. Durant l'étude, 60% avaient été hospitalisés au moins une fois avec un nombre moyen d'hospitalisation de 1,94. La majorité des patients (68,6%) avaient présenté au moins une complication infectieuse au cours de la période d'étude, les infections liées au cathéter étant les plus fréquentes (Tableau 3). Durant la période d'étude de 15 mois, 17 abandons (8,6%) ont été enregistré et 114 patients (58%) sont décédés (Figure 1). La majorité des décès était enregistré chez les patients incidents: à 15 mois, la survie des patients incidents était de 18% contre 43% chez les prévalents (p< 0,001), Figure 2 (A et B). L'urémie, les sepsis sur cathéter et les accidents cardiovasculaires étaient les principales causes décès (Tableau 4). Une courte durée du suivi néphrologique avant la dialyse (r= -0,248, p=0,009), un faible taux de LDL à l'entrée en dialyse (r= -0,385, p=0,007), la survenue d'un AVC (r= 0,188, p=0,011) et l'urémie (r= 0, 299, p<0,001) étaient positivement corrélés au décès. Par contre aucune association n'a été observée entre l'âge, le diabète, le score de Charlson, le statut VIH, le taux d'hémoglobine, l'albuminémie et la mortalité. Les patients prévalents (p<0,001), la résidence dans la même ville que le centre de dialyse (p=0,0033), la prise en charge non familiale (p=0,0446), le suivi avant la dialyse supérieur à 3 mois (p<0,001), le taux de cholestérol total à l'entrée en dialyse supérieur à 1,5 g/l (p=0,0024), un score mental supérieur à 25 (p=0,035) étaient associés à une meilleure survie Figure 2 (B,C,D,E,F,G).

**Tableau 1:** Données sociodémographique et clinique à l’entrée en hémodialyse

**Age** (moyenne en année)	47,97 ± 13,19
35-44	17,8%
45-54	33,5%
55-64	19,8%
**Sexe masculin**	55%
**Emploi** (N=156*)	
Oui	31%
Non	69%
**Prise en charge** (N=156*)	
Personnel	42%
Famille	49%
Etat, assurances, ordre religieux	9%
**Lieu de résidence** (N=156*)	
Identique celui centre de dialyse	109 (70%)
Diffèrent celui centre dialyse	47 (30%)
**Etiologie IRCT** (N=197)	
HTA	35%
Diabète	21%
GNC	20%
HIVAN	7%
Autres ^#^	9%
Inconnue	8%
**Stade MRC au transfert** (N=168)	
1	1(0,5%)
2	0
3	5 (3%)
4	11 (6,5%)
5	151 (90%)
**Durée suivi néphrologique en pré dialyse** (moyenne)	5,58 mois (extrêmes 1-60)
**Compliance au suivi néphrologique en prédialyse** (N=38^&^)	
Bonne	39,5%
Médiocre	21%
mauvaise	39,5%

* données non disponibles chez tous les patients

# polykystose rénale, néphropathies obstructives, NTIC

& évaluée uniquement chez les patients avec suivi néphrologique pré-dialytique > 3 mois

**Tableau 2:** Données paracliniques et comorbidités à l’entrée en dialyse

**Paramètres à l’entrée en dialyse** (moyennes) N= 109*	
Créatininémie (mg/dl)	18,74± 12,26
Azotémie (g/l)	2,44 ± 1,05
Hémoglobine (g/dl)	7,57 ± 1,99
Albuminémie (g/l)	40 ± 1,18
Cholestérol total (g/l)	1,91 ± 0,71
Triglycéride (g/l)	1,25 ± 0,61
HDL (g/l)	0,42 ± 0,17
LDL (g/l)	1,22 ± 0,67
Kaliémie (mmol/l)	5,20± 1,26
Calcémie (mg/l)	78, 75 ± 13,68
Phosphorémie (mg/l)	69,13 ± 31,15
**Comorbidités à l’entrée en dialyse** N=109*	
Score de Charlson (moyenne)	2,14 ± 2,375
Diabète	20,81%
Insuffisance cardiaque	13,73%
VIH	7,1%
Cancer	1,5%
Absence comorbidité	40,1%
**Dialyse programmée** N=197	
oui	9,7%
Non	90,3%

* Evalué uniquement chez patient incidents

**Tableau 3:** Répartition selon les comorbidités survenue pendant le suivi

Paramètres	N	%	Totaux
**Complications infectieuses**			
Infection de cathéter	67	59,3%	
tuberculose	9	8%	
Méningite	3	2,65%	
Infection pulmonaire	9	8%	
Infection urinaire	12	10,6%	113*
Endocardite	3	2,65%	
autres^α^	10	(8,8%)	
**Urémie**			197
oui	121	61,4%	
Non	76	38,6%	
**AVC**			182ᶣ
oui	8	4,4%	
Non	174	95,6%	
**Nombre hospitalisation**			115*
0-1	63		
2-3	46		
≥ 4	6		

* données disponibles chez 107 patients

α autres infections digestives, cutanées et ORL ; les accès palustres n’étaient pas répertoriés

ᶣ données non disponibles chez tous les patients

**Tableau 4:** Causes des décès durant la période de l’étude

Causes de décès	0-3 mois		3-6 mois		7-15 mois		Totaux
	Patients incidents	Patients prévalents	Patients incidents	Patients prévalents	Patients incidents	Patients prévalents	
**Urémie**	26	-	8	14	5	7	60
**Sepsis sur cathéter**	13	-	1	1	-		15
**AVC**	-	4	-	3		1	8
**Infarctus**	-	1	-	-	-	-	1
**Hyper kaliémie**	2	-	1	2	-	-	5
Autres*	11	-	-	4	4	6	25
**Totaux**	52	5	10	24	9	14	114
**Totaux**	57		34		23		

* Autres: anémie, hémorragie digestive, infection non liée au cathéter, indeterminée

## Discussion

L'épidémiologie de l'IRCT traitée varie selon les pays. Dans les pays développés, elle affecte les sujets âgés, les maladies dégénératives telles que le diabète et l'HTA sont les principales étiologies tandis que dans les pays sous-développés, notamment en Afrique sub-Saharienne, elle touche les sujets jeunes (+++ entre 20-50 ans), les glomérulonéphrites sont l'une des principales étiologies et plus de 75% des patients nécessitent le recours à la dialyse à l'arrivée en néphrologie [2]. Ceci est corroboré par nos résultats. En effet, l'âge moyen de nos patients était de 47,97 ans. De même, 90% de nos patients étaient transférés tardivement, avec une durée moyenne du suivi en pré dialyse de 5,58 mois et seulement 9,7% des dialyses étaient programmées. Plus de la moitié de nos patients sont décédés au cours de notre étude, notamment au cours des 3 premiers mois (50% des décès entre 0-3 mois), avec une survie moyenne de 8 mois et une survie à 15 mois de 30,77%; la survie. Ces survies sont plus faibles que celles retrouvées dans les pays développés [3–5]. Cette différence s'explique par des conditions de dialyse plus rudes. En effet, la faible survie dans notre série est la combinaison de multiples facteurs, notamment du transfert tardif, du manque d'adhérence au traitement, des ruptures de matériel de dialyse et des comorbidités. Ainsi, dans notre série, la réapparition des signes d'urémie en dialyse (essentiellement liés au manque d'adhérence et aux ruptures de matériel de dialyse) était la première cause de mortalité, et était corrélé au décès. De plus, la moyenne d'heure en dialyse par semaine était de 6,62, et explique la forte contribution de l'urémie à la mortalité dans notre série, d'autant qu'une cohorte en Lituanie a démontré qu'une durée en dialyse < 8 heures/ semaine était un facteur de risque indépendant de mortalité [6]. Par contre, nos survies sont comparables à celles rapportées dans les pays d'Afrique Subsaharienne. Au Nigeria, Ekrikpo et al retrouvent une survie à 2 ans de 27%, avec comme principal facteur de risque de décès la non adhérence aux séances de dialyse du fait des coûts très élevées [7]. Arogundade et al, à Ile-Ife au Nigeria retrouve que seulement 5% des patients survivent au-delà de 3 mois en dialyse; en effet, le cout élevé de l'hémodialyse explique que seul 5% des patients traités par hémodialyse arrive à se maintenir en dialyse après 3 mois et seuls ces patients survivaient [8]. Des données similaires sont également retrouvées par Ulasi et al avec ¾ des patients qui abandonnent l'hémodialyse principalement pour des raisons financières [9]. De même, Tamiru et al en Ethiopie, dans une cohorte rétrospective de 10 ans, ont retrouvé une survie à 1 an de 42,1% avec une survie moyenne de 263 jours, soit 8,8 mois; le principal facteur prédictif de décès était le type d'abord vasculaire et les cathéters étaient associés à un pronostic médiocre [10] ; reflet probable du transfert tardif qui, dans notre étude était corrélée au décès. Eghan et al au Ghana ont également rapporté une survie de 45% à 3 mois [11]. Ainsi, les survies en hémodialyse dans les pays d'Afrique Subsaharienne semblent assez similaires avec des survies moyennes généralement inferieures à 1 an et la plupart des patients décédant au cours des 3 premiers mois. Ces survies sont le reflet de conditions de dialyse assez similaires: transferts tardifs, non adhérence à la dialyse, souvent liés aux coûts élevés du traitement, saturation des centres disponibles et manque de personnel prédominent [12].

Les sepsis sur cathéter étaient la deuxième cause de décès et prévalaient surtout au cours des 3 premiers mois de dialyse ; ceci témoigne du transfert tardif et de l'absence de suivi en pré dialyse. L'utilisation abusive des cathéters temporaires du fait de l'indisponibilité des permcaths, avec des durées d'utilisation de plusieurs semaines, expliquent également ce résultat. L'importance des infections de cathéter comme causes de mortalité a également été retrouvé par Tamiru et all en Ethiopie [10] et Eghan et al au Ghana [11]. Les complications cardiovasculaires représentaient la 3^ème^ cause de décès de 0-15 mois et la 2eme cause de décès après 6 mois. Dans les pays développés, elles représentent la 1ere cause de décès [3]. Toutefois, ceci démontre que malgré les conditions de dialyse difficiles (coût, transfert tardif, mauvaise adhérence), l'IRCT reste un puissant facteur de risque cardiovasculaire même dans notre contexte. Les patients qui résidaient dans la même ville que le centre de dialyse avaient une meilleure survie que ceux qui résidaient dans des villes différentes. Ce résultat pourrait s'expliquer par une adhérence moins bonne chez ces derniers. Cette mauvaise adhérence est partiellement expliquée par les couts supplémentaires liés au transport. Par ailleurs, l'absence d'adhérence au traitement est un facteur indépendant de mortalité bien décrit en hémodialyse [7, 13]. Contrairement à Mapes et al [14], seul le score mental était corrélé à la survie dans notre série. Cette différence s'explique par le fait que tous nos malades avaient une mauvaise condition physique et traduit l'influence de l'état psychologique sur la survie en dialyse.

**Limite de l'étude:** la durée de l'étude n'était que de 18 mois, il aurait était souhaitable qu'elle soit plus longue. Par ailleurs, la fréquence des ruptures de matériel et leur impact sur la mortalité n'a pas été évaluée.

**Force de l'étude:** il s'agit de la première étude évaluant la morbidité et la mortalité des hémodialysés au Cameroun. De plus, elle incluait tous les patients, incidents et prévalent hémodialysés du pays. Elle fournit ainsi une base pour l'évaluation des nouvelles stratégies de prise en charge instituées depuis 2009 ; entre autre l'augmentation du nombre de centre dialyse (la plupart des régions du Cameroun dispose actuellement d'au moins un centre de dialyse), le meilleur suivi de la disponibilité du matériel de dialyse limitant les ruptures) et l'augmentation du nombre de néphrologues, quoique restant toujours insuffisant. Il est donc indispensable d'entreprendre une autre étude afin de réévaluer la survie des patients, d'apprécier l'impact de ces progrès sur la survie des hémodialysés chroniques et d'évaluer les nouveaux déterminants de la survie en hémodialyse au Cameroun.

## Conclusion

La mortalité en hémodialyse au Cameroun est élevée, avec une survie moyenne de 8 mois et une survie à 3 mois de 47,7%. La plupart des décès sont enregistrés au cours des 3 premiers mois. La sous dialyse, le transfert tardif et la ville de résidence du patient sont les principaux facteurs de risque de décès. Des efforts doivent être entrepris pour favoriser le transfert précoce en néphrologie, améliorer la disponibilité du matériel de dialyse et uniformiser la distribution des centres de dialyse dans les différentes régions du Cameroun.

### Etat des connaissances actuelle sur le sujet

- Aucune donnée antérieure disponible sur ce sujet au Cameroun.

### Contribution de notre étude à la connaissance

- Première étude évaluant la morbidité et la mortalité des hémodialysés au Cameroun ;
- Inclusion de tous les patients, incidents et prévalent hémodialysés du pays;
- base pour l'évaluation des nouvelles stratégies de prise en charge instituées depuis 2009 (augmentation du nombre de centre dialyse, le meilleur suivi de la disponibilité du matériel de dialyse limitant les ruptures et l'augmentation du nombre de néphrologues).
